# A cautionary note on the studies using the picture-word interference paradigm: the unwelcome consequences of the random use of “in/animates”

**DOI:** 10.3389/fpsyg.2023.1145884

**Published:** 2023-05-05

**Authors:** Ana Rita Sá-Leite, Montserrat Comesaña, Carlos Acuña-Fariña, Isabel Fraga

**Affiliations:** ^1^Cognitive Processes and Behavior Research Group, Department of Social Psychology, Basic Psychology, and Methodology, University of Santiago de Compostela, Santiago de Compostela, Spain; ^2^Institut für Romanische Sprachen und Literaturen, Goethe University Frankfurt, Frankfurt, Germany; ^3^Psycholinguistics Research Line, CIPsi, School of Psychology, University of Minho, Braga, Portugal; ^4^Cognitive Processes and Behavior Research Group, Department of English and German, University of Santiago de Compostela, Santiago de Compostela, Spain

**Keywords:** animacy, picture-word interference paradigm, lexical access, animate nouns, inanimate nouns, Animate Monitoring Hypotheiss, language production

## Abstract

The picture-word interference (PWI) paradigm allows us to delve into the process of lexical access in language production with great precision. It creates situations of interference between target pictures and superimposed distractor words that participants must consciously ignore to name the pictures. Yet, although the PWI paradigm has offered numerous insights at all levels of lexical representation, in this work we expose an extended lack of control regarding the variable animacy. Animacy has been shown to have a great impact on cognition, especially when it comes to the mechanisms of attention, which are highly biased toward animate entities to the detriment of inanimate objects. Furthermore, animate nouns have been shown to be semantically richer and prioritized during lexical access, with effects observable in multiple psycholinguistic tasks. Indeed, not only does the performance on a PWI task directly depend on the different stages of lexical access to nouns, but also attention has a fundamental role in it, as participants must focus on targets and ignore interfering distractors. We conducted a systematic review with the terms “picture-word interference paradigm” and “animacy” in the databases PsycInfo and Psychology Database. The search revealed that only 12 from a total of 193 PWI studies controlled for animacy, and only one considered it as a factor in the design. The remaining studies included animate and inanimate stimuli in their materials randomly, sometimes in a very disproportionate amount across conditions. We speculate about the possible impact of this uncontrolled variable mixing on many types of effects within the framework of multiple theories, namely the Animate Monitoring Hypothesis, the WEAVER++ model, and the Independent Network Model in an attempt to fuel the theoretical debate on this issue as well as the empirical research to turn speculations into knowledge.

## 1. Introduction

**Figure 1:**
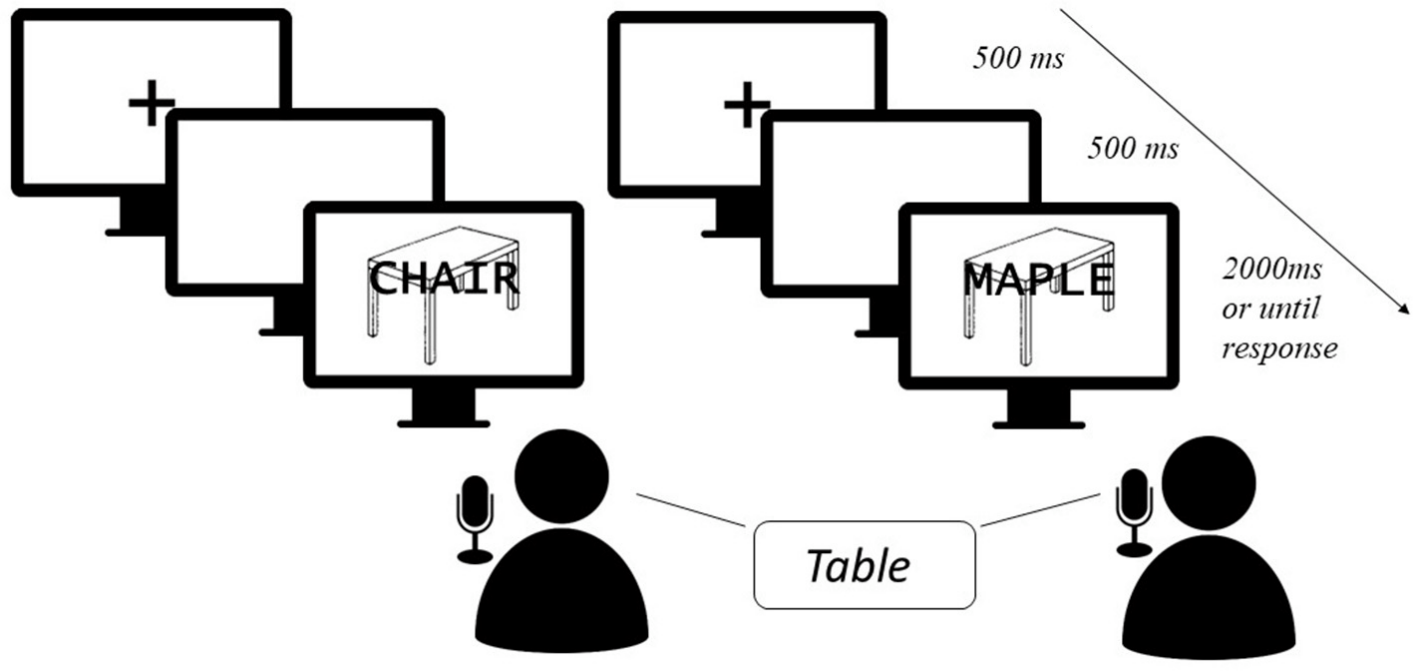
Example of a typical PWI task. In this example, distractors are presented written over the targets, rather than orally. Presentation of both targets and distractors is simultaneous, but different stimulus onset asynchronies have been tested in which the distractor can be presented either before or after the target (and, differently from a prime word, it is maintained on the screen along with the target). In the condition to the left, there is a semantic relationship between target “table” and distractor “chair”; in the condition to the right, there is phonological overlap between target “table” and distractor “maple”. The image of a table was taken from the International Picture Naming Project (IPNP) database (Szekely et al., 2004).

The picture-word interference (PWI) paradigm has served as a window for the study of lexical access at the level of semantics, grammar, and ortho-phonology. It is a variant of the Stroop task in which the classic Stroop effect (Stroop, 1935) is caused by the simultaneous or quasi-simultaneous presentation of images and distractor words that share the linguistic aspects under study (Lupker, 1979; Dell'Acqua et al., 2007; Shao et al., 2015; Starreveld and La Heij, 2017). When confronted with this type of paradigm, participants have to name pictures aloud using either a noun or a short noun phrase whilst ignoring a distractor word, usually a noun, that is either superimposed over the picture or presented auditorily (see Figure 1). The sharing of certain characteristics between target and distractor is expected to affect the response times of the participants. The effects that have been mostly explored are probably the ones concerning semantics and ortho-phonology. Indeed, when both nouns are from the same semantic field, interference is usually obtained (Cutting and Ferreira, 1999). For instance, the picture of an “apple” is generally named faster when paired with the distractor “table” than when paired with the distractor “orange”. Yet, when the semantic relationship is associative, this is, when nouns tend to happen together in speech, such as “dog” and “bone”, facilitation is obtained (Sailor et al., 2009; Geng et al., 2013). As for the so-called phonological facilitation effect, the sharing of the initial or final syllable or last letters/phonemes when stress patterns are controlled facilitates picture naming (e.g., Meyer and Schriefers, 1991; Melinger and Abdel Rahman, 2004; Ayora et al., 2011; Wilshire et al., 2016).

The PWI paradigm hence offers a versatile experimental option in which the interference created by the reading of a written noun during language production gives interesting insights about the way lexical access occurs. For instance, among many other contributions, it offers evidence about how neighboring lexical entries compete during the selection of a certain noun (Alario and Martín, 2010), how grammatical gender is accessed depending on the presence of an agreement context (Cubelli et al., 2005), or how cross-linguistic influence between languages occurs, including the possible interaction between spoken and sign languages (Giezen and Emmorey, 2016). The complementary use of other measuring techniques, namely electroencephalography or fMRI, further extends the evidence obtained with this paradigm by providing information about the temporal and neural organization of lexical encoding (Abel et al., 2009; Bürki et al., 2016). More recently, the PWI paradigm has been used as a resource to understand how lexical access is affected in its different levels in the context of normative aging (Lorenz et al., 2018) and a range of clinical conditions, namely second language impairment (de Hoog et al., 2015), apraxia of speech (Mailend and Maas, 2013), or aphasia (Hashimoto and Thompson, 2010).

However, the most ingenious element of the PWI paradigm, i.e., the use of language comprehension to study language production in its oral and written form (Bonin and Fayol, 2000; Bürki et al., 2019), involves a complex process whose outcomes can be misleading. In the PWI paradigm trials consist of a target that is both a picture and a noun, along with a written or oral distractor noun. This means that during the design of a PWI task, authors have to take into account multiple variables from three different stimuli (that may belong to different modalities, e.g., visual target and its associated noun plus orally presented distractor). Acknowledging this situation is critical because the outcome of a PWI task can be influenced by a great range of uncontrolled variables, not only of a psycholinguistic nature (e.g., the visual complexity of the images, the tone of the oral distractor). Indeed, such degree of complexity can be troublesome and has actually been regarded as a source of possible disruption in the observation of effects (for more detail, see the systematic/meta-analytic reviews of Bürki et al., 2020, and Sá-Leite et al., 2022). In this sense, one variable has been recently pointed out as possibly relevant: animacy (Sá-Leite et al., 2021).

Animacy may be understood as a gradient feature, a continuum in which humans are at one extreme and are followed by other categories such as mammals, other animals, plants, and objects (Dahl, 2000; The Animacy Hierarchy, Aissen, 2003). Across this continuum we can locate a cutoff point whose limits are often vague, but whose presence stablishes a cognitively relevant dichotomy between animate entities (e.g., elephants, jellyfish) and inanimate objects (e.g., tree, table).^1^ Such a dichotomy has shown to have clear cognitive repercussions at attentional, memory-related, and psycholinguistic levels (Rakison and Poulin-Dubois, 2001; Nairne et al., 2017), with multiple studies having shown that there are different brain regions specialized in the processing of either animates or inanimates (e.g., Perani et al., 1995; Mahon et al., 2009; Proklova et al., 2016). The impact of this dichotomy can be observed even in toddlers, since it has shown to be a central organizing principle of children's cognitive experiences (e.g., Rostad et al., 2012).

Coming back to the PWI paradigm, different cognitive processes in which animacy may have an impact are involved in the resolution of the task, namely, the degree of attention given to target and distractor, or the number of semantic features associated with the target and distractor nouns to be accessed. Surprisingly, only Sá-Leite et al. (2021) seem to have considered animacy as a potential intervening factor in the PWI paradigm, specifically when considering the area of grammatical gender encoding. More precisely, the authors analyzed a typical PWI effect, the gender congruency effect, through the scope of animacy. The gender congruency effect consists in modulations on the response times of the participants depending on the gender congruency between the target and distractor nouns. Many authors have combined nouns of different gender (e.g., masculine and feminine) as target-distractor pairs to check whether response times are affected depending on the activation and selection of one gender node or another. Yet, the outcome of these experiments is often mixed, with effects of facilitation being found in both directions (for both gender congruent pairs and incongruent pairs) and with many factors affecting the outcome (for a meta-analytic review, see Sá-Leite et al., 2022). When trying to better understand the gender congruency effect, Sá-Leite et al. (2021) manipulated the number of animate target pictures within the stimuli list and discovered that the effect was only present for the list featuring exclusively inanimate targets. The mere presence of 25% of animate targets prevented registering a significant effect, and the integrative analysis of all stimuli from all lists showed an effect of gender congruency restricted to inanimate targets which was smaller than the effect obtained when only the stimuli from the list with exclusively inanimate targets were considered. This led the authors to wonder what the effect of animacy might be in the activation of gender and to alert other authors regarding the overall role of animacy in the PWI paradigm.

In the present work, we discuss the possibility of animacy having an impact on the general outcome of a PWI paradigm across the different effects under study. Note that the nature of this work is hence speculative and intends nothing more than to nurture a theoretically motivated debate among researchers and hopefully inspire future studies that might turn speculation into possible evidence. With this aim, we first assess the cognitive impact of animacy on the mechanisms of attention, as well as the possible consequences that such an impact can have for the outcomes of a PWI paradigm, and then we do the same regarding the role of animacy in language processing. Afterwards, we present the reader with a systematic review in which we assess the animate status of targets and distractors within the PWI paradigm across all studies. As we will see, animacy has been almost completely ignored either as a confounding or as an independent variable.

### 1.1. The impact of animacy on attention

The most important theoretical framework on the link between animacy and attention was developed by New et al. (2007) under the name of “Animate Monitoring Hypothesis”. The authors conducted a series of change-detection tasks in which both animate and inanimate stimuli were included in pictures of naturalistic scenes that suffered changes. More specifically, participants were rapidly presented with pairs of similar naturalistic scenes (250 ms each), but the second scene suffered changes regarding the presence or absence of animate and inanimate stimuli in relation to the first scene. The results showed that participants were faster and more likely to detect changes in animate than inanimate stimuli. The authors explained these results as a matter of ancestral priorities: the experience of humans living during millennia in hunter–gatherer environments would have derived in the ontogenetical development of an attentional advantage for animacy.

These ideas were supported by numerous studies (New et al., 2010; Yang et al., 2012; Altman et al., 2016; but see Hagen and Laeng, 2016; He and Cheung, 2019), even with toddlers (Hofrichter et al., 2021). Altman et al. (2016) study is especially interesting because they conducted change-detection tasks but analyzed in more detail not only the performance on specific stimuli but the influence of the presence of these stimuli on the detection of changes in others. The results not only showed the typical animacy advantage, but also showed that the detection of changes in inanimate stimuli is hampered by the presence of animate stimuli in the scene, but not vice-versa. This was true even when these animate stimuli remain unchanged and camouflaged. Similar outcomes were obtained in other paradigms. Visual search tasks also showed that animate entities are located faster than inanimate objects (Jackson and Calvillo, 2013). In particular, Calvillo and Hawkins (2016) observed that both threatening and non-threatening animate entities were more frequently detected than their inanimate object counterparts. Likewise, Guerrero and Calvillo (2016) conducted an attentional blink task with animate and inanimate stimuli. Attentional blink refers to the phenomena by which participants fail to detect the second target in a task in which two target items are presented very closely in time (~500 ms) in a series of rapid presentations. Their results were clear: animate targets were detected significantly more times than inanimate targets, and hence they were less prone to experience the phenomenon of attentional blindness. Ro et al. (2007) conducted multiple experiments in which participants searched for a green frame among blue frames. More specifically, they were asked to make speeded categorical decisions on stimuli presented within the green target frame (e.g., “was it food?”). Their results showed clearly that animate stimuli were attended preferentially (Ro et al., 2007). Animate stimuli are also detected more frequently than inanimate items in situations of both low and high perceptual load (Calvillo and Jackson, 2014), and animate motion is detected more quickly than inanimate motion (Pratt et al., 2010), even for newborn infants (Di Giorgio et al., 2017, 2021).

In sum, it seems undeniable that there is an attentional advantage when it comes to animate stimuli, whose presence seems to negatively affect the perception of inanimate stimuli. This can have important consequences for the outcomes of a PWI experiment, especially considering the inclusion of animate stimuli as target pictures. Thus, not only would the mechanisms of attention prioritize these stimuli over others, this is, over the distractors, but also the perception of a distractor would be especially hampered by the mere presence of an animate target (e.g., “elephant”). This could mean that the “distracting” role of the distractors is at least partially attenuated when animate targets are included. Since their potential to interfere decreases, competition between animate targets and inanimate distractors (“elephant” - “pencil”) would produce smaller effect sizes than in the case of inanimate targets and distractors (“house” - “pencil”). On the other hand, when distractors are animate their ability to interfere should increase. On a pure attentional basis, an effect size should hence be greater for purely animate target-distractor pairs (“elephant” - “king”) than for animate target/inanimate distractor pairs (“elephant” - “pencil”). Similarly, the increased ability to interfere of an animate distractor (“king”) should create even greater competition with an inanimate target (“house”), powering even more the effects of competition in comparison to animate target/animate distractor pairs (“elephant”/”king”) and inanimate target/inanimate distractor pairs (“house”/”pencil”). In any case, understanding the effect of animate nouns on the outcomes of PWI experiments is a topic that must also be addressed from the perspective of Psycholinguistics, as assessed in our next section.

### 1.2. How animacy impacts lexical access

Evidence suggests that animate words are somehow privileged during lexical access. For instance, animate targets are consistently named faster than inanimate targets (Laws and Neve, 1999; Laws et al., 2002). Even though we could think that this advantage at naming tasks could be explained by the attentional bias we discussed in the previous section, evidence shows that the existing differences in the performance of participants when considering animate vs. inanimate nouns cannot be exclusively explained on the basis of such a bias (see Xiao et al., 2016). In this sense, the reasons behind this advantage are usually related to the semantic content of animate nouns, as they are considered semantically “richer” than inanimates. This has been explained in multiple complementary ways that are backed up by numerous studies. Among these explanations, a commonly cited one is the theory that animate nouns present greater overlap among them in terms of semantic features, by which is meant that animates are overall more similar to each other than inanimates (e.g., Cree and McRae, 2003; Zannino et al., 2006; Davis et al., 2014; Xiao et al., 2016). Indeed, animates form categories of words that are semantically closer than those of inanimates and whose activation shows highly similar brain patterns (Xiao et al., 2016). Other studies suggest that animate nouns have more sensorimotor features than inanimate ones (Hargreaves et al., 2012a; Bonin et al., 2014; Heard et al., 2019). This is because animate nouns are related to more sensory and/or perceptual experiences than inanimates (Bonin et al., 2014). Indeed, words associated with more sensorimotor features have been found to be better recalled and recognized as well as processed faster as a function of their lexico-semantics (Hargreaves et al., 2012b; Hoffman et al., 2013). To be precise, animate nouns have shown to be consistently better recalled and recognized than inanimate ones (Nairne et al., 2013, 2017; VanArsdall et al., 2013, 2014). Regarding lexico-semantic encoding, an advantage of animate nouns over inanimate nouns has been found in semantic/animacy categorization tasks and lexical decision tasks as well (e.g., Becker et al., 1997; Radanović et al., 2016; Bonin et al., 2019).

Although many studies observe the advantage of animate nouns over inanimate nouns and obtain evidence regarding the semantic richness of animacy, another proposal offers an interesting and empirically supported view on the mechanism behind this advantage, i.e., why being semantically richer (greater overlap, greater number of sensorimotor features) translates into faster response times or better accuracy. It is based on the concept of *lexical accessibility*. If we define language production as an incremental process by which speakers can begin to generate speech once minimal input is made available (and hence word class, number, gender, phonological form, orthographic form, etc. are encoded incrementally and in parallel), how each piece of linguistic information is processed depends on its own relative accessibility. In this sense, information that is retrieved easily is given priority over information that is retrieved less easily. The relative ease of information retrieval depends on the baseline levels of activation of the information to be encoded. For instance, the relative accessibility of the elements of a syntactic structure has been shown to depend on whether or not these have been activated earlier through previous production or comprehension (Branigan et al., 2000). In this regard, animacy has been recognized as one of the factors that impact the relative accessibility of conceptual information (i.e., conceptual accessibility: the number of pathways available for retrieval, so that the more the pathways to the lexical concept, the faster its retrieval; Bock and Warren, 1985). Concepts that refer to animates would therefore be faster retrieved for production than those that refer to inanimates. This would be related to a semantic dimension they call *predicability* (Bock, 1987), this is, the number of conceptual relations an entity can establish. Animates can establish many more conceptual relations than inanimates. For instance, a dog can be born, bought, adopted, abandoned, it can die, sleep, communicate, bark, attack, run, walk, sit, break things, get hurt, be scared, etc., whilst the number of conceptual relations of a table or even a plant are much more reduced. Thus, animates not only tend to have more semantic overlap between each other and more sensorimotor features, but they also tend to be more predicable than inanimates and hence to enter in more syntactic relations (a tendency that can be broken for certain examples, e.g., bacteria, which is animate vs. doll, which is inanimate). This entails a higher conceptual accessibility. In sum, we could say that animates have a rich semantic content that contributes to an increase of their baseline activation level relative to inanimates. Therefore, they have a higher conceptual accessibility because they are prioritized by our system and are hence retrieved faster than inanimate nouns.

**Figure 2:**
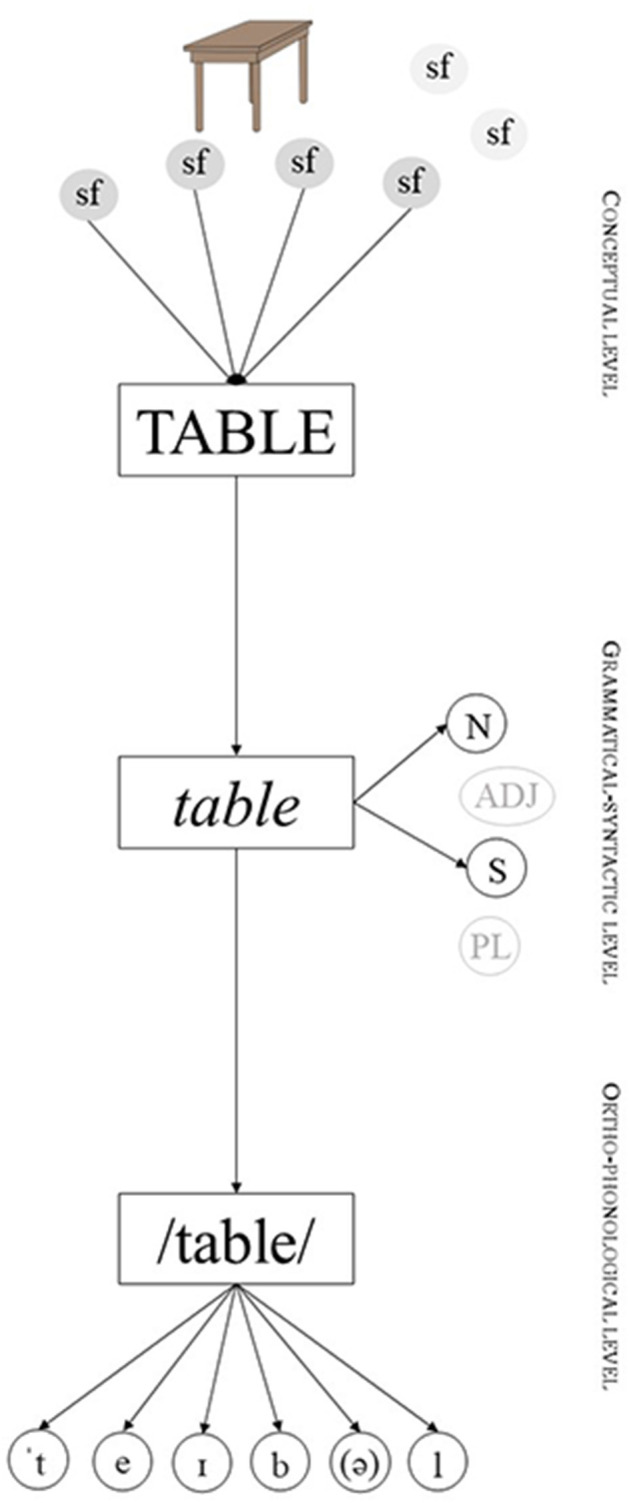
Simplified structure of lexical access during the production of the noun “table”. Semantic features with lighter background at the conceptual level are meant to represent other features that are not related to “TABLE” and hence are not active. The same applies to lighter features at the grammatical-syntactic level. Arrows represent the flowing of activation. Sf, Semantic Feature; *N*, Noun; Adj, Adjective; S, Singular; PL, Plural. Based on the architecture proposed by WEAVER++. Adapted from Levelt et al. (1999).

To better understand the consequences of the semantic peculiarities of animate nouns in lexical access and hence, in the PWI paradigm, let us first introduce the typical structure of lexical access as proposed by most models of language processing, including the influential Word-form Encoding by Activation and VERification model ++ (WEAVER++) (Roelofs, 1992, 1993; Levelt et al., 1999). In simple terms, three types of information must be encoded when accessing a noun: conceptual information related to meaning, grammatical-syntactic information, and form-based information (see Figure 2). All these three types of information are organized following three levels of lexical representation formed by nodes. Thus, for instance, the noun “table” is defined by a specific set of semantic features (e.g., “furniture”, “four legs” “wood”, “place to eat/work”, etc.) represented by nodes at the conceptual level of representation. When producing “table”, all these nodes are activated in the speaker's lexicon. This activation then spreads to the other levels of representation, namely, the grammatical-syntactic level in which features such as word class (noun) and number (singular) are activated and selected, and the orthographic-phonological level in which the phonological representation of the word is encoded (e.g., /'teıb(ə)l/). This forms a pattern of activation that specifically represents the word to be produced, in this case, “table”. Importantly, when the word “table” is not to be produced, it remains at a basal level of activation [lower level of activation than the required for production (or recognition) to occur]. This baseline level of activation can be higher the more we use the word (the basal level of activation depends on the frequency of use of a word - it is higher for “table” than for instance “cacophony”) or depending on other factors impacting conceptual accessibility (e.g., animacy).

Now, note that in light of the literature that we have just reviewed on animacy, the semantic particularities of animate nouns may have direct repercussions on the effects obtained with the PWI paradigm, mainly those of semantic nature. Indeed, it is well known that the higher the semantic overlap between the two stimuli, the stronger the competition for selection between both lexical entries (as both are highly activated and reinforce each-other), and the greater the semantic relatedness effect. In this sense, whilst inanimate nouns do not share many semantic features simply because of being inanimate (e.g., “car” and “pencil” are both inanimate but highly different), animates not only tend to have a higher number of semantic and sensorimotor features than inanimate nouns, but they also tend to share a vast amount of these features. Take, for instance, “gorilla” and “zebra”: both are alive, both are animals, both are mammals, both have eyes, both have teeth, both are viviparous, both are hairy, both live in the outside, both are vegetarian, both have a heart, both feel pain, both have a nervous system, and so on. From this, we may speculate that the strongest effect of semantic competition within a PWI paradigm would be obtained when both target and distractor are animates. Importantly, the semantic overlap that animates *naturally* have could have general consequences for the outcome of a PWI paradigm, as facilitative and competitive effects of other types may behave differently for pure animate target-distractor pairs, in which the primary source of interaction between both entries is of semantic competition. Consequently, not only semantic relatedness effects should be analyzed through the scope of animacy, that is, taking into consideration possible differing size effects for semantically similar animates pairs in comparison to semantically similar inanimate pairs, but also pure animate pairs should be considered with caution when studying other type of effects.

The fact that animate nouns are semantically richer ultimately means that the number of semantic features to be processed at the conceptual stage of lexical access is greater than that of inanimate nouns. This points to the idea that our system may devote a great number of cognitive resources to animate nouns than inanimate nouns, particularly when considering the semantic level of lexical encoding. When animate nouns are being comprehended and produced, more resources would have to be devoted to process the semantic information of animates – perhaps to the detriment of other levels of encoding, something that has been called semantic prioritization (Sá-Leite et al., 2021). We could hence think that semantic prioritization can affect the amount and distribution of cognitive resources across the other levels of lexical encoding. The grammatical level of lexical encoding might be in a particularly fragile position. This is because it would be especially prone to suffer the possible consequences of high amounts of semantic information needing to be processed while the speed of lexical access still has to be increased for the sake of animacy itself. Indeed, the WEAVER++ model highlights the idea that grammatical information is selected (fully encoded) only when necessary – this leaves the door open to the idea that grammatical information does not have to be *always* selected. Another classic model of language production, the Independent Network model (Caramazza, 1997), remarks that grammatical encoding can be skipped as it is not a compulsory intermediate step between semantics and word-form encoding. Thus, on these views, language production can occur with information flowing directly from the semantic to the ortho-phonological level. In short, the idea that grammatical information can be skipped under certain conditions is not new and fits in with the evidence and theories on animacy. Indeed, it would seem as if our cognitive system devoted a higher number of cognitive resources to process the greater amount of semantic information of animate nouns, but we were still faster processing them in comparison to inanimate nouns. If grammatical processing can be skipped for specific reasons, animacy might perhaps be one of those reasons, so that semantics can be prioritized whilst maintaining lexical access especially fast. This theory is in line with the results of Sá-Leite et al. (2021), who systematically failed to observe effects based on gender processing for animate nouns, as if grammatical gender were not being encoded in these cases. Since grammatical gender is not an indispensable characteristic to be encoded when an agreement context is not present [which is the case of Sá-Leite et al. (2021) study, whose participants only produced bare nouns (i.e., with no adjectives or determiners whose form co-changes with the form of the noun)], once the system is overflown by the processing of animacy but is still forced to prioritize the processing of animate words, it would seem as if it dropped grammatical gender from the processing stage. Yet, this is a speculative hypothesis that should be further tested experimentally. As suggested by a Reviewer, one way of testing this would be designing an experimental situation in which the number of resources available was manipulated (e.g., a concurrent task manipulation). If the gender congruency effect requires a certain amount of available resources in order to emerge, the effect should disappear if the task is made more difficult with a concurrent task draining some of those resources away.

Finally, facilitative effects based on orthography and phonology could theoretically suffer variations due to the presence of animate stimuli as well. Note that facilitation here means that the distractor is speeding up the processing of the target, probably by contributing to the activation of the shared word-form attributes. Thus, if the target is animate (“baboon”) and the ortho-phonologically related distractor is inanimate (“typhoon”), the facilitative effect produced by the inanimate distractor could be particularly small. This is because the target is already being processed quite fast and it is perhaps maintained at a high basal level of activation by our system. In fact, maybe we should consider the possibility of a ceiling effect for animate targets. The size of the phonological effect would hence decrease in comparison to pairs formed by an inanimate target. On the contrary, the combination of an inanimate target (“vanilla”) and an animate distractor (“gorilla”) would increase the size of the effect due to an accentuated facilitative role by the animate distractor, which would be highly and quickly activated. Now, if both target and distractor are animate, there is a potential confound with the strong effect of semantic relatedness we mentioned before, and hence we are not sure of how facilitative effects of phonological overlap would behave in this scenario.

### 1.3. Summing up the interference of animacy on the PWI

Taking into consideration the impact of animacy on the human attentional processes, in general, any effect of interaction between target and distractor could be influenced by animacy in the following way: animate targets will hamper the perception and interfering/facilitative role of inanimate distractors, diminishing the observed effects; animate distractors will have an increased interfering/facilitative role when paired with inanimate targets, increasing the observed effects. Purely animate pairs would hold stronger effects than animate target-inanimate distractor pairs.

On the other hand, we propose that the semantic particularities of animate nouns would have a main role on the outcome of a PWI paradigm, overruling any attentional bias in the case of studies exploring the semantic relatedness effect, grammatical effects, or to a certain degree, orthographic and phonological facilitation effects. Regarding the semantic relatedness effect, the strongest competition should occur between targets and distractors that are animate due to the semantic prioritization of both nouns and to the high degree of semantic similarity. Regarding grammatical effects, if the grammatical aspects at issue are skippable (such as gender with no agreement context), effects on their basis may not even be observed when one of the stimuli is animate due to semantic prioritization. Regarding orthographic and phonological facilitation effects, the main point to have into consideration is the fact that the overall degree and speed of activation of animate nouns is higher than that of inanimate nouns. Ultimately, this could mean that animate targets will benefit less from the presence of an ortho-phonologically similar distractor regardless of its animacy status; contrariwise, an inanimate target will benefit to a higher extent from the presence of an ortho-phonological similar animate distractor in comparison to a similar inanimate distractor.

Further speculating about how attentional and semantic factors interact to predict the outcome of a PWI task is out of the reach of a theoretical paper such as this one. The same applies when trying to understand how the semantic factors of animacy would affect other linguistic effects, such as the semantic association effect (Brooks et al., 2014), the word-frequency effect (Mulatti et al., 2015), or the compound effect (Lorenz and Zwitserlood, 2016).

## 2. The present study

It seems clear that animacy is an important factor in the organization, structuring, and functioning of our cognitive system, with important attentional repercussions as well as consequences at different levels of language processing. It is therefore possible that animate stimuli pose a source of disruption in the outcomes of experiments done with the PWI paradigm, and they might have an interesting role if considered within the experimental design, especially for effects of a semantic nature. Yet, the question remains: how many studies using this paradigm have controlled or considered animacy? In the next section, we will present a systematic review of this matter in detail.

### 2.1. Systematic search

**Figure 3:**
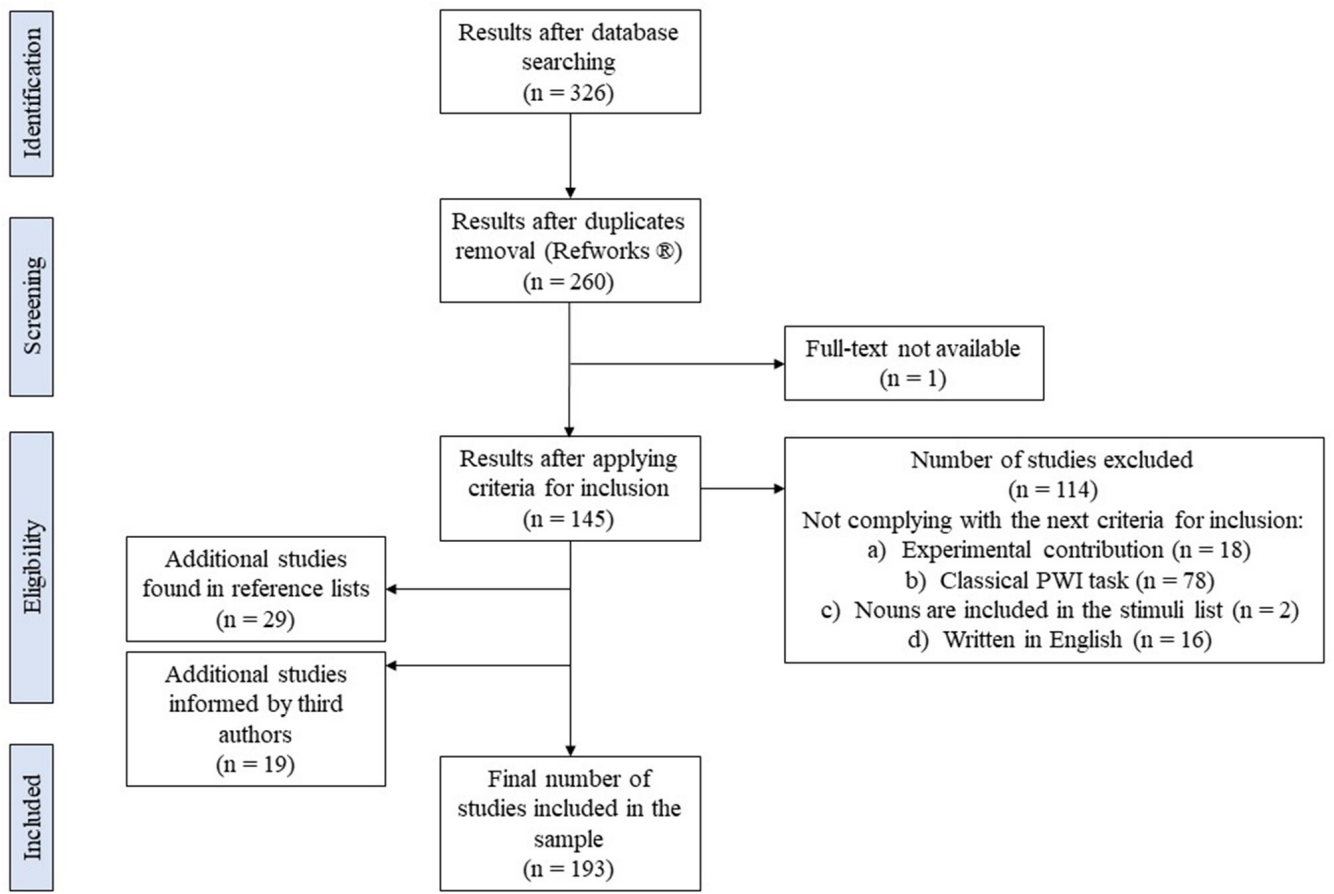
Structure of the search according to the Preferred Reporting Items for Systematic Reviews and Meta-analyses (PRISMA) flowchart of the literature search (Moher et al., 2009).

We conducted a search with the keyword “picture-word interference paradigm” by itself as well as combined with the keyword “animacy” in the databases PsycInfo and Psychology Database. The whole process of systematic search is summarized in the PRISMA graph presented in Figure 3. Our search cast a total of 326 results. After removing duplicates with the software RefWorks^®^ (*n* = 66) a total of 260 studies remained. We checked for availability of the full text of all the studies. When we lacked the permission to access the full text online, authors were contacted mainly through ResearchGate (e.g., Bürki and Madec, 2022). We could not find or obtain upon request the full text of one of the studies (Collina et al., 2014). All the remaining 259 studies were inspected and the next criteria for inclusion were applied:

a. The study makes an experimental contribution (e.g., Mahon and Caramazza, 2009; Sá-Leite et al., 2019, 2020; Fuhrmeister and Bürki, 2022; i.e., it is not a commentary, a theoretical proposal, a systematic review, or a meta-analysis).
b. The study includes at least one PWI task which is not a variation of the classic task (e.g., using a post-cue naming paradigm,^2^ Hocking et al., 2010; Mädebach et al., 2018; using a picture-sound interference paradigm).^3^
c. The study uses nouns or noun phrases that include nouns as either targets or distractors or both (e.g., they do not use verbs as both targets and distractors; Lüttmann et al., 2011).
d. The study is written in English (Yu and Shu, 2003; e.g., a few of the studies were exclusively written in Chinese: see Qingfang and Yufang, 2004).

After applying the criteria for inclusion, 114 studies were disregarded. The inspection of the reference list of each one of them (*n* = 145) allowed us to obtain 29 new studies not contemplated in the initial search that complied with the criteria of inclusion (check the Supplementary Materials for the full list of additional studies). By considering a previous systematic review on the Stroop task and the PWI paradigm by MacLeod (1991), we obtained a total of 19 PWI studies that fitted our criteria for inclusion and were published before 1990. A total of 193 studies were kept in the final sample.

### 2.2. Inspection of animacy

All 193 works were inspected independently by two evaluators with knowledge of gender processing and animacy. A description of the works was made according to: (a) the effect being explored; (b) whether or not animacy is explicitly mentioned and considered; (c) whether or not animacy is considered as a potential confounding factor; (d) examples of animate stimuli target-distractor pairs. To do so, both evaluators first assessed whether or not the paper considered animacy theoretically in the Introduction; then, regardless of whether the study did or did not mention animacy in that section, they assessed whether the paper considered animacy in the Method, namely in the control of the materials, the design, or the results. To conclude, the Discussion was assessed in case the authors decided to consider it at the end as a *post-hoc* explanation of the results, a limitation, or a future research step. Finally, they inspected the stimuli list when available (either within the paper or as an online appendix). When the stimuli list was unavailable, the evaluators checked the examples provided in the Method section. The assessments of both evaluators were compared. When any of the information did not match, the work in question was checked again. The details of every study are collected in Table 1 available at https://doi.org/10.17605/OSF.IO/CJR37.

### 2.3. Summary and description of the studies

The close inspection revealed that from a total of 193 works, only 12 did not mix animate and inanimate stimuli randomly within the target and distractor pairs and the experimental conditions (Ehri, 1976; Guttentag and Haith, 1979; Schnur et al., 2006; Foucart et al., 2010; Muehlhaus et al., 2013; Hwang and Kaiser, 2014; Dank and Deutsch, 2015; DiBattista, 2015; Shin, 2016; Bürki et al., 2019; Deutsch and Dank, 2019; Sá-Leite et al., 2021). Additionally, among these 12, only three of them explicitly stated that they controlled animacy (Foucart et al., 2010; Shin, 2016; Bürki et al., 2019; i.e., “only inanimate stimuli were used”) and only one included animacy as a factor to check its impact on the effect sizes (Sá-Leite et al., 2021). Importantly, only Shin (2016) and Sá-Leite et al. (2021) explicitly mention and discuss animacy theoretically as a potential factor affecting the results. Four studies controlled or manipulated animacy as a factor, but this was not due to animacy itself but because animacy is at the core of certain grammatical cut-offs that happened to be the object of study (Hwang and Kaiser, 2014; Dank and Deutsch, 2015; Deutsch and Dank, 2019; e.g., natural vs. grammatical gender). Guttentag and Haith (1979) decided to only use animate nouns and distractors to study the memory capacity of their participants using the PWI paradigm – they did not mention animacy, though. Ehri (1976), who studied the general mechanisms of attentional interference through the PWI paradigm, was cautious when deciding to use only pairs of semantically related targets and distractors, so that animals were only paired with animals, to avoid any type of confounding effect. Finally, among the 12 studies there is one Thesis (DiBattista, 2015) which considered the impact of animacy theoretically on certain effects obtained with other types of tasks, but not within the PWI paradigm itself – however, the author included only inanimate stimuli in the experiment featuring the PWI paradigm. None of the studies inspected the possible role of the different degrees of animacy (i.e., the animacy hierarchy).

The remaining 181 studies failed to control (explicitly or not), consider, or even mention animacy. All these studies hence feature uncontrolled animate/inanimate target-distractor pairs e.g., when studying phonological overlap and grammatical gender, target “leopard” with distractor “brother-in-law” and target “leopard” with distractor “clay” seen as comparable as target “pear” with distractors “tie” and “beaver”, Bürki et al., 2016). Around 50% of them explore effects of a semantic nature (e.g., semantic relatedness, semantic association, etc.) and still fail to take animacy into account, even though animacy has a direct impact on the semantic richness and the conceptual availability of the noun, as well as on the number of shared features between nouns of the same semantic category. For instance, Rosinski (1977) studies the semantic relatedness effect and considers two categories, animals and household objects, but fails to take the opportunity to check whether the category animals holds stronger effects of semantic interference than that of household objects. Overall, situations arise in which the authors assume the size of the effect of semantic relatedness to be the same for pairs such as “chair_(target)_-sofa_(distractor)_” and “frog-cat” (Collina et al., 2013). They also compare semantically related pairs to unrelated pairs even if animacy is probably undersizing/oversizing the interaction between target and distractor in the unrelated condition. For instance, in one study the pair formed by “frog” and “pen” was compared to the pair formed by “chair” and “child” (Collina et al., 2013). Certainly, despite the fact that both pairs are from the unrelated condition, the attention given to “frog” is probably higher than that given to “chair” and the interference from “child” is probably far higher than the interference from “pen”. In this line, the intrusion of animates of different degrees of animacy within the condition of semantic unrelatedness is quite widespread. We can find semantically unrelated pairs such as “pear-sheep” or “mouse-brush” that are put at the same level as “dog-truck” or “bench-wolf” (Melinger and Abdel Rahman, 2004; Janssen et al., 2008; Jerger et al., 2013; Krott et al., 2019; Jescheniak et al., 2020). In terms of comparisons of effect sizes across semantic conditions, another interesting example is that of De Zubicaray et al. (2013). The authors compare the size effects between conditions of semantic relatedness and semantic association. Yet, they do this without taking into account animacy, which derives in situations in which the *semantic association* is made between an animate and an inanimate, such as “baby” and “pram”, and compared to the *semantic relatedness* between two animates such as “baby” and “priest”. They thus do not ponder the possibility that rather than differences between types of semantic relations, they may be also observing differences due to the animacy of the distractor. The same applies when they compare the semantic association between an inanimate (e.g., “cave”) and an animate (“bat”) to the semantic relatedness of two inanimate nouns (“cave” and “sea”).

Other types of effects we highlighted as especially prone to suffer from interference due to animacy were of a grammatical nature. From the 193 studies, 40 studied some type of grammatical effect (grammatical class effect, case status effect, classifier and gender congruency effect, countability congruency effect), and 6 belong to the 10 that did not mix animate and inanimate stimuli in an uncontrolled manner (still, 3 of them happened to control animacy due to its role as a cut-off point for their object of study, and not due to animacy itself). Therefore, among these studies, we observe situations in which the authors compare conditions with a different number of animate stimuli (gender congruency 13, gender incongruency 7, Schiller and Caramazza, 2003; gender incongruency + semantic relatedness, 4; gender incongruency + semantic relatedness, 0), as well as many random pairs, such as “ax-emperor” belonging to one condition but “ax-rhythm” to the opposite. A curious example is the interesting study by Fieder et al. (2018), which explores the processing of count and mass nouns but assumes that the incongruent (in terms of countability) pairs “kings-yogurt” and “nuns-sand”, to be the same as “pedals-vinegar” (Fieder et al., 2018).

Regarding effects of orthographic and phonological facilitation, besides the random inclusion of mixed pairs, it is interesting to see the use of animate pure pairs without considering the fact that there is a great semantic overlap in that case and hence the effect of phonological facilitation is probably interacting with an effect of semantic interference. For instance, pairs such as “pig” (target) and “rabbit” (Costa et al., 2003), “penguin” and “farmer” (“penguin” was paired with “pizza” in the phonologically unrelated condition; Ayora et al., 2011), “dog” and “goat” (“dog” was paired with “dot” in the related condition, Roelofs and Verhoef (2006), and so on. In addition to the studies exploring semantic, orthographic, phonological, and grammatical effects, the rest of the literature also presents many examples of mixed animate/inanimate pairs. For instance, Dhooge and Hartsuiker (2011) explore frequency effects, and hence compare the times to name low and high frequency pictures depending on whether they are paired with either low or high frequency distractors. Yet, they do not control for animacy, which means that they have combinations such as low frequency animate pictures (“fox”) paired with high frequency animate distractors (“king”), and high frequency inanimate pictures (“stone”) also paired with the same high frequency animate distractors (“king”). In the case exemplified between parentheses, “fox” may be more protected against the interference generated by “king” than inanimate nouns of low frequency, and thus the comparison with “stone” is not as precise as it should. Also, “king”, as a human animate noun, would be an especially interfering distractor in comparison to inanimate nouns. In this line, Geng et al. (2014) also assess the naming times depending on whether the targets are paired with high or low frequency distractors. However, by not considering animacy, the authors create situations in which the high frequency distractor for “drum” is “woman”, but for “hat” it is “air” and for “pig” it is “name”; all this whilst the low frequency distractor for “drum” is “bacon”, for “hat” it is “owl” and for “pig” it is “bale” (among many other examples). Likewise, Schnur et al. (2006) asked their participants to use short sentences to name pictures in which different people were performing different actions. The authors were particularly interested in the effect of phonological relatedness that could emerge between the verbs used in the target sentences and the distractor nouns. However, even though all their pictures depicted humans and all their distractors were inanimate nouns (e.g., dam, dish, jug, rust...), a possible undersizing of the expected effect due to the animacy of the targets was not discussed. Furthermore, some studies used pseudowords as distractors, and assumed the potential interfering role of these strings of letters to be the same both when paired with animate (“farmer”, “mouse”) and inanimate targets (“house”, “needle”, e.g., Oppermann et al., 2008; Brooks et al., 2015). It is also interesting to see how certain studies exploring the perceptive and attentional mechanisms of humans by manipulating the type, position, and other characteristics of the distractor, also disregarded animacy and did not consider differential effects depending on the animacy of targets and distractors (e.g., Underwood, 1976; Toma and Tsao, 1985).

Finally, out of the 181 studies that did not consider or control animacy, 11 involve major works (doctoral theses) and, importantly, 17 explore clinical or aging populations, whose results can have important repercussions regarding our understanding of these conditions. Indeed, it should not be a problem if populations are compared but still confronted with the same set of stimuli (e.g., deaf children, children with Specific Language Impairment, and hard-of-hearing children, de Hoog et al., 2015). Yet, by unknowingly obtaining underestimated or overestimated effects we may be missing precision when judging the specific capabilities of each group - also, semantic processing may be somehow especially impaired in certain clinical populations, which may have a special impact on animacy. In this sense, a relevant example is that of Durfee (2019), who assesses language impairment after a stroke through the size of the effects of semantic relatedness and phonological overlap without considering animacy. This can therefore lead to quite imprecise results, especially if both effects are compared to determine the affection of each of the language abilities.

## 3. Discussion

In the present study, we discussed the possible role of animacy in the outcomes of experiments done using a specific task: the PWI paradigm. Indeed, the PWI paradigm is a vastly used task for the assessment of language processing at the different levels of lexical encoding. However, it involves a complex interaction between comprehension and production processes in which three stimuli of different types (target picture, target noun, written or oral distractor noun), can be sources for confounding variables. We theorized that animacy might be especially relevant both as a possible confounding variable and an independent variable for the outcome of a PWI paradigm because it has great repercussions on (a) the monitorization of attention and hence on the degree of attention given to the target picture and the distractor noun, and (b) on language processing itself, by determining the amount and overlapping of semantic information to be processed and the distribution and number of resources implied in each stage of lexical access. Yet, our systematic review of the literature using the PWI task has shown that animacy has been mostly neglected when it comes to both the control of the materials and its direct study as an independent variable. Of a total of 193 studies reviewed, only 12 have managed to control for animacy. Three of them have done so explicitly, four of them have done it indirectly because animacy is at the base of the cutoff points for the grammatical structures under study, two of them have done it indirectly by controlling the category of “animals”, one of them actually included animacy as a design factor, and the other two happened to use only inanimate stimuli. The remaining have mixed together animate and inanimate stimuli from different points of the animacy continuum, without any regard for the experimental conditions. Among them, a few are of clinical orientation and thus establish conclusions on the language capabilities of populations with clinical conditions affecting language and lexical access.

The apparent absence of animacy in such a vast portion of the literature using the PWI paradigm comes as rather surprising to us. This is because, as hypothesized in the Introduction, the inclusion of animate stimuli can overestimate or underestimate the obtained effects within the PWI paradigm, and can also give interesting insights regarding lexical access in terms of semantic processing, the mandatory processing of grammatical features, or the distribution of resources during the different stages of lexical encoding. In attentional terms, animacy may have a role on the general outcome of a PWI paradigm by maximizing or minimizing the interfering role of the distractors. From the point of view of language processing, animacy is quite interesting as a factor *per se* since its semantic peculiarities might affect specific effects differentially and exploring it may give researchers insights about the way cognitive resources are distributed across the different stages of lexical access. In this sense, regarding the semantic interference effect, the competition between pure animate target-distractor pairs might be especially strong in comparison to semantically similar inanimate pairs due to a greater number of semantic and sensorimotor features and a greater overlap between them. Yet, none of the reviewed studies has considered this. On the other hand, semantic prioritization may somehow affect how cognitive resources are distributed at the other levels of lexical encoding. Of interest is the impact that animacy may have at the level of grammatical encoding. This is because, in line with previous models of lexical access, grammatical encoding has been said to be skippable (Caramazza, 1997; Levelt et al., 1999), thus putting the effects of a grammatical nature in a particularly fragile position. More exactly, if cognitive resources are directed to the conceptual level of encoding and lexical access has to be quick for the sake of animacy, the skipping of grammatical encoding, when possible, might be a useful way of effectively distributing and preserving cognitive resources while speeding up word processing. Finally, effects of orthographic and phonological facilitation may also be affected by the degree and speed of activation of animate nouns. More specifically, the processing of an animate target noun would perhaps not benefit so much from the presence of an ortho-phonologically similar distractor, but an inanimate noun would benefit to a higher extent from the presence of an ortho-phonological similar animate distractor than of an inanimate distractor. Importantly, in lexical terms, the semantic characteristics of animates may affect other specific effects of different lexical nature, especially when including pure animate target-distractor pairs to study other effects such as those of orthographic and phonological facilitation, in which the semantic interference effect coming from the overlap of animate characteristics in the pure animate target-distractor pairs should not be ignored. Still, these are all mere speculations raised to create debate among researchers and which necessarily would have to be put under test. Should authors test any of these ideas, they would inevitably also have to carefully consider whether the results of PWI experiments inform us on the deployment of attentional mechanisms or on semantic prioritization, or both (and, if both, when and how). A disentanglement between both type of impacts (attentional vs. linguistic) could be better explored with additional techniques, mainly fMRI, which could show the differential activation of areas related to attention and linguistic processing among the different types of stimuli. Electroencephalographic techniques would also be interesting to have an idea of the time-course of lexical access, for instance to detect effects of semantic interference in pure animate pairs when studying effects of orthographic and phonological nature.

In sum, we hope that this work captures the attention of researchers when it comes to animacy, as we believe there is enough empirical evidence to think that animacy might have the potential to be a fruitful variable for the PWI paradigm. Of course, in terms of experimental control, we are aware that neglecting animacy as a confounding variable in some cases probably does not have a great impact on the results of a study and the conclusions to be drawn. Some imprecision from mixing animate and inanimate stimuli in an uncontrolled manner might arise, but this imprecision still does not change the final results. After all, for manipulations in which the same stimuli are used in different conditions, the same amount of animates are present of each condition, and the research interest is on the impact of those conditions on the interference effect. Still, we believe that the present work makes a point that is important in the current state-of-the-art: highlighting that most PWI studies are ignoring a variable that has enough theoretical foundation to be considered of high interest for the PWI task due to the characteristics of the paradigm itself.

## Author contributions

AS-L: idea, theoretical background, writing, discussion of the results, systematic review, and funding. MC: revision and systematic review. IF: revision and funding. CA-F: revision, editing, and funding. All authors contributed to the article and approved the submitted version.

## Footnotes

^1^Following the definition proposed by Rakison and Poulin-Dubois (2001), animate entities are self-propelled, their line of trajectory is smooth, can cause action at a distance, their pattern of interaction is contingent and tend to be the agents of action (e.g., “elephant”, “gardener”, “fish”), whilst inanimate objects' motion is caused by external agents, their motion is usually irregular, they do not cause action at a distance by themselves, only by contact, their pattern of interaction is noncontingent and they are the recipient of actions rather than their agent (e.g., “tree”, “table”, “car”; for more details on this definition see Rakison and Poulin-Dubois, 2001).

^2^Within this variation of the PWI paradigm, both target and distractor are pictures, and the target picture is cued subsequently to its presentation along with the distractor picture. For instance, the target could be presented in green tones, and the distractor in blue tones. A subsequent cue (e.g., a green dot) tells the participant which picture to name.

^3^In the picture-sound interference paradigm, the distractor is a sound rather than a written word. For instance, a semantic interference effect within this paradigm would arise from presenting the picture of a dog with the sound of a dog barking in comparison to the sound of a car engine.
